# Methylation estimates the risk of precancer in HPV-infected women with discrepant results between cytology and HPV16/18 genotyping

**DOI:** 10.1186/s13148-019-0743-9

**Published:** 2019-10-12

**Authors:** Rubí Hernández-López, Attila T. Lorincz, Leticia Torres-Ibarra, Caroline Reuter, Dorota Scibior-Bentkowska, Rhian Warman, Belinda Nedjai, Indira Mendiola-Pastrana, Leith León-Maldonado, Berenice Rivera-Paredez, Paula Ramírez-Palacios, Eduardo Lazcano-Ponce, Jack Cuzick, Jorge Salmerón, FRIDA Study Group, Attila Lorincz, Cosette Wheeler, Patti Gravitt, Eduardo Lazcano, Leticia Torres, Leith León, Paula Ramírez, Berenice Rivera, Eduardo L. Franco, Jack Cuzick, Pablo Méndez, Jorge Salmerón, Mauricio Hernández, Anna Barbara Moscicki, Yvonne Flores, Enrique Carmona, Kathleen M. Schmeler, David Bishai, Pilar Hernández, Rubi Hernández, Indira Mendiola

**Affiliations:** 1 Centro de Investigación en Salud Poblacional, Instituto Nacional de Salud Pública, Cuernavaca, Morelos México; 2 Centro de Investigación en Políticas, Población y Salud, Universidad Nacional Autónoma, Ciudad de México, México; 3 Wolfson Institute of Preventive Medicine, Centre for Cancer Prevention, Queen Mary University of London, London, UK; 4 Cátedra-CONACYT, Centro de Investigación en Salud Poblacional, Instituto Nacional de Salud Pública, Cuernavaca, Morelos México; 5 Unidad de Investigación Epidemiológica y en Servicios de Salud, Delegación Morelos, Instituto Mexicano del Seguro Social, Cuernavaca, Morelos México

**Keywords:** DNA methylation, S5 classifier, Human papillomavirus, *EPB41L3*, Triage, Cervical intraepithelial neoplasia, Cervical cancer

## Abstract

**Background:**

Vigilant management of women with high-risk human papillomavirus (hrHPV) is necessary in cancer screening programs. To this end, we evaluated the performance of S5 (targeting DNA methylation in HPV16, HPV18, HPV31, HPV33, and human gene *EPB41L3*) to predict cervical intraepithelial neoplasia grade 2 or higher (CIN2+) in a sample of hrHPV-infected women referred to colposcopy in the FRIDA Study, a large screening trial in Mexico. A nested case-control sample with women referred to colposcopy either by atypical squamous cells of undetermined significance or higher (ASCUS+) in cytology and/or positive for HPV types 16 or 18 was tested by S5. Seventy-nine cases of CIN2+ were age-matched to 237 controls without a diagnosis of CIN2+ (<CIN2). DNA from exfoliated cervical cells was bisulfite converted and PCR amplified for S5 targets, and methylation was quantified at specific cytosines by pyrosequencing.

**Results:**

The S5 classifier separated women with CIN2+ from <CIN2 with a highly significant area under the curve (AUC) of 0.75 (95% CI 0.69–0.82), while AUC for CIN3+ was 0.81 (95% CI 0.74–0.89). To optimize sensitivity and specificity for Mexico, an alternative S5 cutoff of 3.7 was implemented to account for overall higher methylation seen in our already triaged women. All three invasive cancers were detected by methylation or HPV16/18 but none by cytology. Sensitivity of S5 for CIN2+ was 62% (95% CI 50.4–72.7%), specificity was 73% (95% CI 66.9–78.5%), and adjusted PPV was 15.1% (95% CI 12.0–18.3%). In contrast, the crude sensitivity of HPV16/18 detection and cytology were 63.3% (95% CI 51.7–73.9%) and 57.0% (95% CI 45.3–68.1%) respectively; specificity was 29.1% (95% CI 23.4–35.3%) and 62.4% (95% CI 55.9–68.6%) respectively, while adjusted PPV was 6.4% (95% CI 4.9–8.1%) and 10.5% (95% CI 8.0–13.1%), respectively. Methylation testing could reduce colposcopy referrals by 30 to 50% with virtually no loss of sensitivity for CIN2+ and CIN3+.

**Conclusions:**

S5 testing on hrHPV-positive women significantly increased diagnostic information compared to triage by HPV16/18 plus cytology and appears to have clinical utility as an additional test to substantially lessen burdens on colposcopy.

**Trial registration:**

The FRIDA Study is registered in ClinicalTrials.gov, number NCT02510027.

## Background

Cervical cancer is the fourth leading cause of cancer-related death in women worldwide, and developing regions carry the greatest burden of this disease. In Mexico, cervical cancer is ranked second as a cause of death from malignancy in women [1–4]. The recognition of persistent infection with high-risk human papillomavirus (hrHPV) as a major cause of cervical cancer [5–8] has favored the development of new technologies for hrHPV detection. The introduction of these tests as part of primary screening for cervical cancer in various countries has greatly improved the detection of precancerous lesions [9–12]. Some new testing procedures represent a breakthrough in screening [9, 13, 14]; however, hrHPV testing programs must incorporate triage before colposcopy referral because of the low specificity of hrHPV assays [15, 16]. The use of triage tests should enable the relatively accurate identification of the small proportion of hrHPV-positive women who need to go to colposcopy given their increased risk of developing cancer. Triage can reduce over-diagnosis and over-treatment of clinically non-relevant hrHPV infections [3, 17–19]. Various types of triage tests have been proposed, including cytology, p16/Ki67 immunocytochemistry, and HPV16/18 detection, but these methods still have important limitations such as subjectivity of interpretation, low sensitivity, and low positive predictive value (PPV) [15, 16, 20].

DNA methylation, the enzymatic addition of a methyl group to the carbon at position 5 of the cytosine ring in a cytosine-phosphate-guanine (CpG) site, has been shown to play an important role in the development of the carcinogenic process [21–25]. Many studies have found a strong association between the methylation of host and viral genome with the development of CIN2, CIN3, and cancer [22, 23, 26–29]. Indeed, various quantitative combination methylation assays are currently under evaluation with the most common host genes being *EPB41L3*, *MAL*, *CADM*, *FAM19A4*, and *MIR124*, as well as CpG sites in the late regions of various HPV genomes [24, 30–35]. According to Clarke et al., methylation across all 12 carcinogenic HPV types is associated with precancer (CIN3+), providing a better sensitivity than cytology as triage test [36]. Other methylation marker panels designed from methylation-specific real-time PCR assays are now being validated with promising results [37], in particular, the QIAsure methylation test, which is a multiplex real-time methylation-specific PCR [38], and the GynTect assay based on the detection of DNA methylation of human marker gene regions [37–39]. In our previous studies, we have consistently found that methylation assays targeting the most carcinogenic HPV types help to distinguish progressing infections and more severe lesions [22, 27, 32]. Indeed, we have found that the performance of methylation to detect precancer improves as more HPV viral types and human genes are added [30, 40]. In particular, the development of a methylation score—called S5—combining DNA methylation levels of HPV16, HPV18, HPV31, HPV33, and the human tumor suppressor gene *EPB41L3* has arisen as a promising biomarker panel that can be used to triage hrHPV positive women. At 90% sensitivity, S5 provided a specificity of 49% to identify or predict the risk of a high-grade cervical lesion in a UK colposcopy referral population [31]; in other words, one half of the women sent for colposcopy (regarded as an invasive examination) could have been spared costs, a biopsy, and anxiety while being reassured of highly sensitive detectability if they had a precancer. The S5 classifier was developed in a colposcopy study [31] and validated in screening studies in the UK and Canada [28, 32]. The aim of our study was to extend validation of the diagnostic performance of S5 and to find new applications in geographically and ethnically different women undergoing triage in a large population-based HPV screening cohort in Mexico. In this study, we explored the utility of methylation testing in women with discrepant results between cervical cytology and HPV16/18 genotyping. We give the term “already-triaged” to the women in our study to avoid possible confusion over the role of an additional methylation test.

## Results

**Fig. 1:**
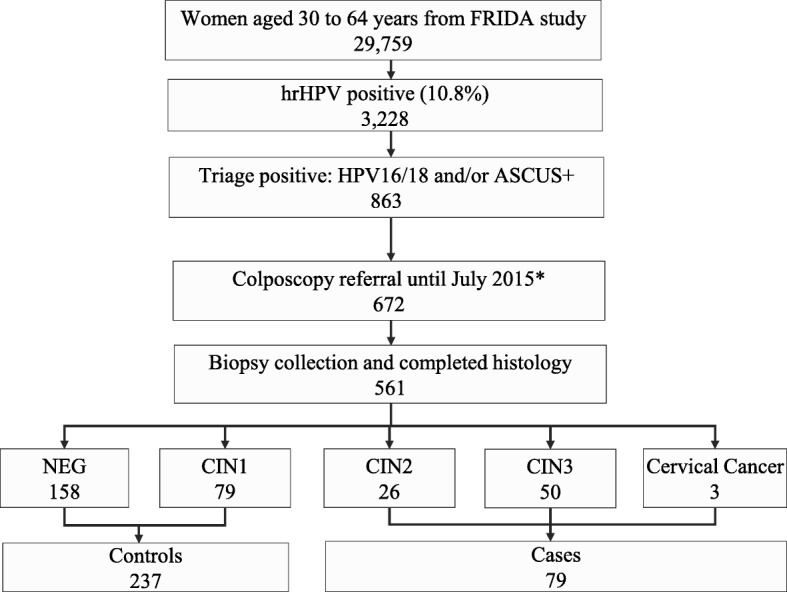
Consort diagram of FRIDA nested case-control triage study showing the numbers of women in each step. Triage positive women included HPV16/18 positive (<ASCUS) (*n* = 508), ASCUS+ (HPV16/18 negative) (*n* = 277), and HPV16/18 positive and ASCUS+ (*n* = 78). *Prior to July 2015, 672 out of 863 triage-positive women attended the colposcopy evaluation. Then, 561 women out of 672 who underwent colposcopy had histology results recorded from April 2013 to the time of the study cutoff date on July 15. These 561 women are represented in our sampling frame, from which we selected all the CIN2+ cases (79) and a random selection of three controls per case matched by age (1 CIN1 and 2 NEG). In total, 316 participants were included in our methylation analysis: 79 CIN2+ cases, 79 CIN1, and 158 NEG. The cases included all the CIN2+ detected until July 2015. The three controls per case were randomly selected and matched by age from women with histological diagnoses of CIN1 or less. The remaining 245 triage-positive women with histology results of CIN1 and negative would have been controls but were not selected by the sampling method as we already had adequate power for the study. Abbreviations: hrHPV, high-risk human papillomavirus; HPV16/18, human papillomavirus type 16 or type 18; ASCUS+, atypical squamous cells of undetermined significance or worse; NEG, histologically negative; CIN, cervical intraepithelial neoplasia (of grades 1, 2, and 3)

The 79 cases and 237 controls were composed mostly of women aged between 30 and 49 (92.4%), with a median age of 37 years. Figure 1 shows the consort diagram of our nested case-control study: 29,759 cervical specimens were tested for hrHPV (Cobas 4800). The grouped prevalence of 13 types of hrHPV plus HPV66 (now regarded as a low-risk HPV) was 10.8%. From 3228 hrHPV-positive women, 863 (26.7%) were positive on initial triage (HPV16/18+ and/or ASCUS+); 672 of these women were referred to colposcopy, and 561 attended for colposcopy prior to our nested case-control study cutoff date of July 2015; the colposcoped women were the subjects of our methylation study.

There were no significant differences with respect to demographics and lifestyle characteristics between cases and controls (Additional file 1). Twenty-seven percent of cases and 30% of controls began their sexual life before the age of 16. The majority of cases and 51% of controls reported having had one lifetime sexual partner. The proportion of women who had used hormonal contraceptives for more than 12 months was the same (12.7%) in both groups. Most women in both groups reported not having used a condom during the last 12 months (75% cases and 74% controls). 48.1% of cases and 36.7% of controls had four or more pregnancies before FRIDA enrollment. Overall, 90% of the study population had never smoked (89.8% cases and 93.3% controls).

Focusing on the HPV results, 60.8% of cases and 46.8% of controls were HPV16-positive (*p* = 0.038). However, only 6.3% of cases as compared to 26.6% of controls were HPV18-positive (*p* = 0.000). With respect to other hrHPV types, 24.1% of cases and 19.4% of controls were positive for HPV31 (*p* = 0.422). Similarly, 6.3% of cases and 5.9% of controls were HPV33-positive (*p* = 1.00). Fifty-five percent of both cases and controls were positive for other types of hrHPV plus HPV66. With respect to cytology, 57% of cases and 38% of controls showed ASCUS+ (*p* = 0.004) (Table 1).

**Table 1:** hrHPV type-specific prevalence and cytological evaluation in cases and controls

	Controls (NEG/CIN1) *n* = 237		Cases (CIN2+) *n* = 79			
	Number	Percent	Number	Percent	*p* value^	OR (95% CI)
hrHPV positivity*						
HPV 16+	111	46.8	48	60.8	0.038	1.8 (1.05–2.94)
HPV 33+	8	3.4	3	3.8	1.000	1.1 (0.32–4.04)
HPV 31+	28	11.8	10	12.7	0.843	1.1 (0.51–2.31)
HPV 18+	47	19.8	1	1.3	0.000	0.1 (0.0–0.30)
Other hrHPV+**	43	18.1	17	21.5	0.511	1.0 (0.60–1.67)
Cytological evaluation						
Normal	148	62.5	34	43.0	0.004	0.5 (0.27–0.76)
ASCUS+	89	38.0	45	57.0	0.004	2.2 (1.32–3.68)
LSIL+	74	31.2	41	51.9	0.001	2.4 (1.42–3.99)
HSIL+	10	4.2	22	27.9	0.000	8.8 (3.98–19.3)
HPV and cytology						
HPV16/18+ and ASCUS+^a^	24	10.3	16	20.3	0.030	2.3 (1.14–4.47)
HPV16/18+ and LSIL+^b^	17	7.2	14	17.7	0.014	2.8 (1.32–5.89)
HPV16/18+ and HSIL+^c^	5	2.1	5	6.3	0.128	3.1 (0.94–10.4)
HPV16/18+ and normal^d^	148	62.5	34	43.0	0.004	0.5 (0.27–0.76)
HPV16/18− and ASCUS+^e^	65	27.4	29	36.7	0.121	1.5 (0.90–2.62)

*ASCUS+* atypical squamous cells of undetermined significance or worse, *HSIL+* high-grade squamous intraepithelial lesion or worse, *LSIL+* low-grade squamous intraepithelial lesion or worse, *NEG* histologically negative, *CIN* cervical intraepithelial neoplasia (of grades 1, 2, and 3)

*Ranking of hrHPV genotypes according to the positive predictive values for CIN2+ (Cuzick, 2016)

**Other hrHPV: HPV35, 39, 45, 51, 52, 56, 58, 59, or 68 and HPV66 (a presumptive low-risk type)

**^***p* value of two-sided Fisher´s exact test to evaluate differences in hrHPV prevalence as well as cytology results between cases and controls

^a^Includes HPV16/18+ and ASCUS, LSIL, HSIL, and cervical cancer. ^b^Includes HPV16/18+ and LSIL, HSIL, and cervical cancer. ^c^Includes HPV16/18+ and HSIL and cervical cancer. ^d^Includes HPV16/18+ and normal cytology. ^e^Includes HPV16/18− and ASCUS, LSIL, HSIL, and cervical cancer. Categories a, b, and c are not mutually exclusive

**Fig. 2:**
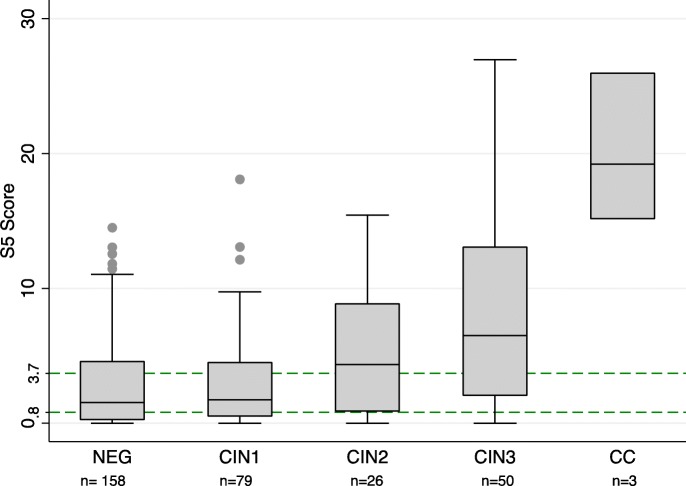
Comparison of S5 methylation classifier in histologically negative (NEG), CIN1, CIN2, CIN3, and cervical cancer cases (CC). The S5 classifier was significantly different between the following group comparisons: NEG vs CIN2 (*p* = 0.01), NEG vs CIN3 (*p* < 0.001), NEG vs CC (*p* < 0.001), CIN1 vs CIN2 (*p* = 0.03), CIN1 vs CIN3 (*p* < 0.001), CIN1 vs CC (*p* = 0.004), CIN2 vs CC (*p* = 0.007), and CIN3 vs CC (*p* = 0.02). Other comparisons were not significant (NEG vs CIN1 and CIN2 vs CIN3). Abbreviations: NEG, histologically negative; CIN, cervical intraepithelial neoplasia (of grades 1, 2, and 3); CC, cervical cancer. The top of box represents the upper quartile (p75), bottom the lower quartile (p25), and the line the median (p50). The upper whisker extends to the largest point of the inter-quartile range from the upper quartile. The lower whisker extends to the smallest point of the inter-quartile range from the lower quartile. The outliers are plotted as individual points for each lesion grade. The Cuzick test for trend was highly significant (*p* < 0.001)

The S5 classifier showed a highly significant increase proportional to the severity of lesions (Cuzick test for trend, *p* < 0.0001). Median methylation was 1.3 in histopathologically negative samples (NEG), 1.7 in CIN1, 4.4 in CIN2, 6.5 in CIN3, and 19.2 in cervical cancer (CC) (Additional file 2). Figure 2 shows the distribution of the S5 classifier by histopathological diagnosis. The Mann-Whitney *U* test revealed highly significant methylation differences in the following pairwise comparisons: NEG vs CIN2 (*p* = 0.01), NEG vs. CIN3 (*p* < 0.001), NEG vs CC (*p* < 0.001), CIN1 vs CIN2 (*p* = 0.03), CIN1 vs. CIN3 (*p* < 0.001), CIN1 vs CC (*p* = 0.004), CIN2 vs CC (*p* = 0.007), and CIN3 vs CC (*p* = 0.02).

**Fig. 3:**
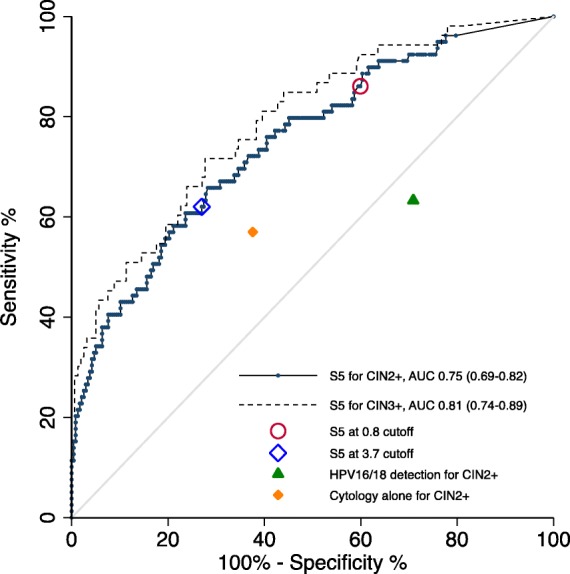
Receiver operator characteristic (ROC) and area under the curve (AUC) of S5 methylation for detecting CIN2+ or CIN3+. The blue diamond denotes the sensitivity and specificity of S5 at a cutoff of 3.7 for CIN2+. The red circle denotes the sensitivity and specificity at the S5 cutoff of 0.8 predefined for use in the UK for CIN2+. The cutoff for cytology alone was ASCUS+. Abbreviations: ASCUS+, atypical squamous cells of undetermined significance or worse

ROC analysis of the S5 classifier for detecting CIN2+ and CIN3+ gave areas under the curve (AUC) of 0.75 (95% CI 0.69–0.82) and 0.81 (95% CI 0.74–0.89) respectively (Fig. 3). A new S5 cutoff value of 3.7 was selected for better discriminating CIN2+ lesions from <CIN2 diagnoses in our already-triaged Mexican women with abnormal cytology and/or HPV16/18 infection (Fig. 3). This cutoff yielded a relative sensitivity and specificity of 62.0% (95% CI 50.4–72.7) and 73.0% (95% CI 66.9–78.5) respectively for CIN2+ (Table 2). The same cutoff for discriminating CIN3+ from <CIN3 produced a sensitivity of 70.3% (95% CI 56.7–81.8) and a specificity of 76.6% (95% CI 70.7–81.9). In addition, we calculated the performance of the S5 classifier with a cutoff of 0.8 which was previously validated in the UK and Canadian screening populations. We observed a better sensitivity for CIN2+ of 86.1% (95% CI 76.5–92.8) (*p* = 0.0005), but a worse specificity of 40.1% (95% CI 33.8–46.6) (*p* < 0.0001) at the 0.8 S5 cutoff.

**Table 2:** Performance of the S5 methylation classifier, cytology, and HPV16/18 genotyping for detecting CIN2+ and CIN3+

	Sensitivity (95% CI)	Specificity (95% CI)	Unadjusted PPV (95% CI)	Adjusted PPV* (95% CI)	Unadjusted NPV (95% CI)	OR (95% CI)
S5 cutoff 3.7						
CIN2+	62.0 (50.4–72.7)	73.0 (66.9–78.5)	43.4 (34.0–53.0)	20.3 (16.7–23.9)	85.2 (79.6–89.8)	4.20 (2.59–7.54)
CIN3+	70.3 (56.7–81.8)	76.6 (70.7–81.9)	45.0 (35.0–55.3)	18.0 (14.3–21.7)	90.5 (85.0–94.2)	7.75 (4.21–14.3)
S5 cutoff 0.8						
CIN2+	86.1 (76.5–92.8)	40.1 (33.8–46.6)	32.4 (26.1–39.2)	13.8 (11.5–16.0)	89.6 (82.2–94.7)	4.14 (2.10–8.14)
CIN3+	92.5 (91.8–97.9)	38.8 (32.9–45)	23.3 (17.8–29.6)	9.9 (8.0–11.9)	96.2 (90.6–99.0)	7.76 (3.83–21.2)
HPV16/18 and cytology for CIN2+						
HPV16/18+	63.3 (51.7–73.9)	29.1 (23.4–35.3)	22.9 (17.5–29.1)	9.0 (7.2–10.9)	70.4 (60.3–79.2)	0.71 (0.42–1.21)
ASCUS+	57.0 (45.3–68.1)	62.4 (55.9–68.6)	33.6 (25.7–42.2)	14.4 (11.5–17.3)	81.3 (74.9–86.7)	2.20 (1.3–3.7)
LSIL+	51.9 (40.4–63.3)	68.8 (62.5–74.6)	35.7 (26.9–45.1)	15.6 (12.3–18.8)	81.1 (75.0–86.3)	2.38 (1.42–3.99)
HSIL+	27.8 (18.3–39.1)	95.8 (92.8–98.0)	68.8 (50.0–83.9)	42.3 (34.0–50.6)	79.9 (74.8–84.4)	8.70 (3.9–19.2)

*ASCUS+* atypical squamous cells of undetermined significance or worse, *HSIL+* high-grade squamous intraepithelial lesion or worse, *LSIL+* low-grade squamous intraepithelial lesion or worse, *NEG* histologically negative, *CIN* cervical intraepithelial neoplasia (of grades 1, 2, and 3).

*Adjusted PPV = (Sn × Pr)/((Sn × Pr) + (1 − Sp ) × (1 − Pr)), where Sn is sensitivity, Sp is specificity, and Pr is the CIN2+ or CIN3+ screening prevalence in the FRIDA population-based study (10% and 6.8% respectively)

Of the 316 triage-positive samples, 218 were positive for HPV16/18 and 134 were ASCUS+ (32 were HSIL). The unadjusted sensitivity of HPV16/18 genotyping for detecting CIN2+ was 63.3% (95% CI 51.7–73.9%), with a specificity of 29.1% (95% CI 23.4–35.3, Table 2). Cytology alone with a cutoff of ASCUS+ had an unadjusted sensitivity of 57.0 (95% CI 45.3–68.1) and an unadjusted specificity of 62.4% (95% CI 55.9–68.8), while cytology with a cutoff of HSIL+ had an unadjusted sensitivity and specificity of 27.8 (95% CI 18.3–39.1) and 95.8% (95% CI 92.8–98.0) respectively.

We investigated the positivity of different triage tests for NEG, CIN1, CIN2, CIN3, and CC histopathological diagnoses (Table 3). In the context of cervical cancer screening in Mexico, a greater specificity would reduce the number of women called for colposcopy (Table 3). For example, HPV16/18 genotyping was positive in 72.2% of histologically negative women as opposed to 27.2% positive with S5 classifier at a cutoff of 3.7. All three cancers were identified by HPV16/18 detection and by the S5 classifier, but all were missed by cytology.

**Table 3:** Positivity of different triage tests for histologically negative (NEG), CIN1, CIN2, CIN3, and CC

	NEG (*n* = 158) % (95% CI)	CIN1 (*n* = 79) % (95% CI)	CIN2 (*n* = 26) % (95% CI)	CIN3 (*n* = 50) % (95% CI)	CC (*n* = 3) % (95% CI)
HPV16/18+	72.2 (64.6–78.6)	68.4 (57.1–77.8)	65.4 (44.3–81.8)	60.0 (45.5–72.9)	100
ASCUS+	30.4 (23.6–38.1)	51.9 (40.7–62.9)	61.5 (40.7–78.9)	58.0 (43.6–71.2)	0
LSIL+	25.9 (19.7–33.4)	41.8 (31.2–53.1)	61.5 (40.7–78.9)	50.0 (36.0–64.0)	0
HSIL+	3.2 (1.3–7.4)	6.3 (2.6–14.6)	19.2 (7.7–40.4)	34.0 (21.9–48.6)	0
S5 at 3.7 cutoff	27.2 (20.8–34.8)	26.6 (17.9–37.6)	50.0 (30.5–69.5)	66.0 (51.4–78.1)	100
S5 at 0.8 cutoff	57.6 (49.7–65.4)	64.6 (53.2–74.5)	73.1 (51.7–87.3)	92.0 (79.9–97.1)	100

*ASCUS+* atypical squamous cells of undetermined significance or worse, *HSIL+* high-grade squamous intraepithelial lesion or worse, *LSIL+* low-grade squamous intraepithelial lesion or worse, *NEG* histologically negative, *CIN* cervical intraepithelial neoplasia (of grades 1, 2, and 3), *CC* cervical cancer

**Fig. 4:**
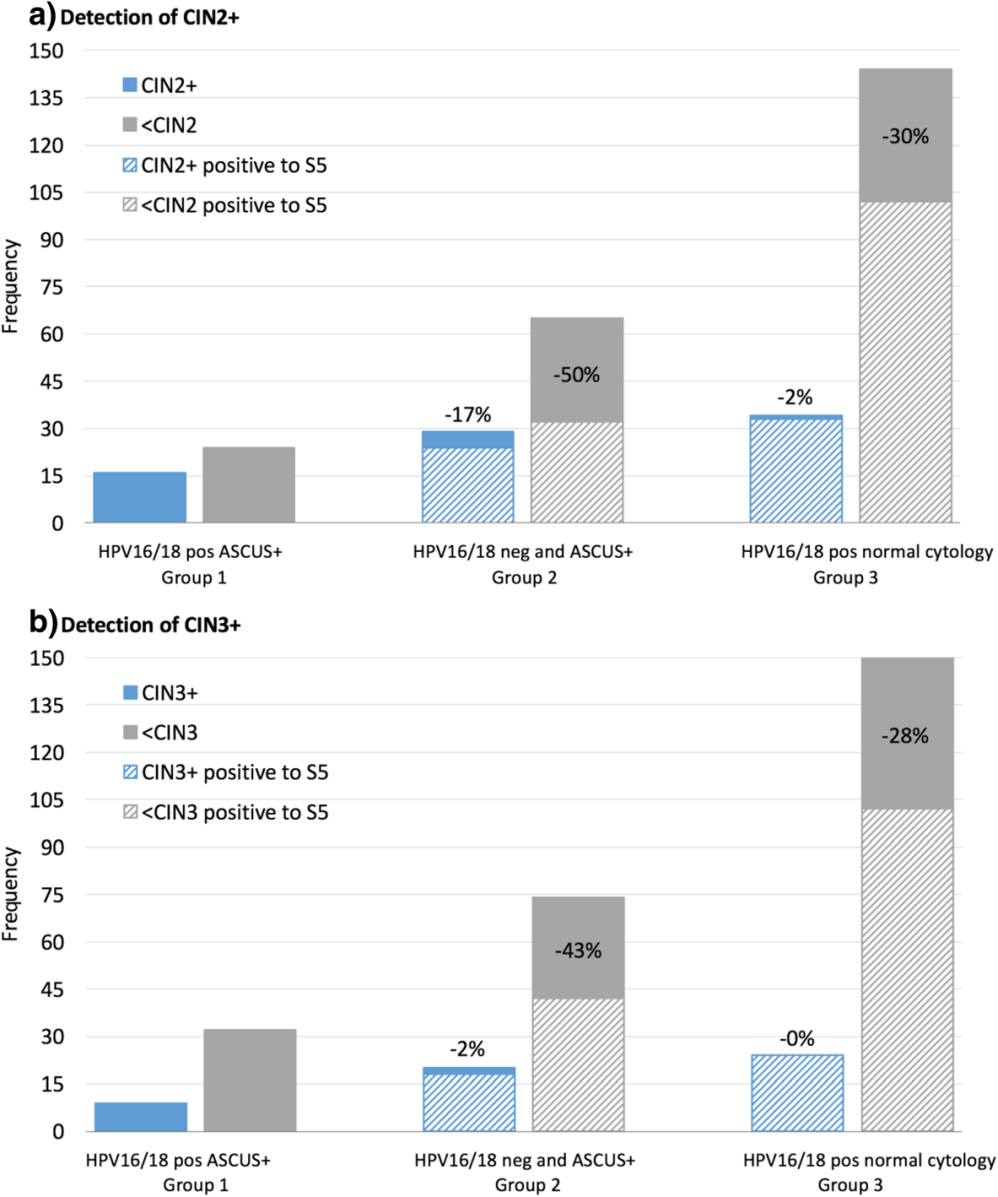
Benefit of using the S5 classifier as a second triage test for colposcopy referral for **a**) CIN2+ and **b**) CIN3+ endpoints. S5 helped reduce unnecessary colposcopy referrals in both the “HPV16/18 neg, ASCUS+” and “HPV16/18 pos, normal cytology” groups. Under the current Mexican algorithm, all HPV16/18 positive and/or ASCUS positive are referred to colposcopy, but we propose to use S5 as second triage test for the discrepant triage groups (“HPV16/18 neg, ASCUS+” and “HPV16/18 pos, normal cytology”) which then decreases the false-positive rate. We therefore defined the following hypothesis to test S5 benefits. In group 1, women were HPV16/18 positive and ASCUS positive and must be referred to colposcopy without any additional triage procedure. In group 2, women were HPV16/18 negative and ASCUS positive and were called for colposcopy, but if we had used S5 methylation as a second triage test, it would have reduced by 50% (CIN2 endpoint) or 43% (CIN3 endpoint) the number of false positives referred to colposcopy. Using S5 methylation as a second triage for women in group 3, who were HPV16/18 positive with normal cytology, would have reduced unnecessary colposcopy referrals by 30% in CIN2+ and 28% in CIN3+. Abbreviations: ASCUS+, atypical squamous cells of undetermined significance or worse; CIN, cervical intraepithelial neoplasia (of grades 1, 2, and 3). The frequency shows the absolute number of women in each group

We subsequently explored the clinical utility of the S5 methylation classifier as a second or reflex triage test (Fig. 4). The women in our study had increased risk of CIN2+ whether they were positive for either or both ASCUS and HPV16/18; therefore, we defined three risk groups of women based on the outcomes of the latter two tests. The first group consisted of HPV16/18-positive and ASCUS-positive women. We consider that these women should be referred to colposcopy based on the first triage alone. A second group represented a triage discrepancy consisting of women negative for HPV16/18 but positive for an ASCUS+ result. In our study, these women were also called for colposcopy, but in retrospect, some of them were considered to have gone unnecessarily. The use of S5 methylation as a second triage test in this group would have reduced colposcopy referral by 50% for a CIN2+ endpoint (*p* = 0.0000) or 43% for a CIN3+ endpoint (*p* = 0.0000). In the third group, women were HPV16/18 positive but had normal cytology. All these women were also called for colposcopy. Here again, the use of S5 methylation would have reduced colposcopy referral by 30% (CIN2+ endpoint, *p* = 0.0000) (Fig. 4a) or 28% (CIN3+ endpoint, *p* = 0.0000) (Fig. 4b). In both groups 2 and 3, the sensitivity of S5 as the second triage test was statistically no different than in a scenario where all women were sent to colposcopy (Table 3 and Fig. 4). This means that using S5 as a reflex triage test would reduce the number of women unnecessarily sent to colposcopy by 30 to 50% while maintaining the same sensitivity to detect CIN2+ (or CIN3+) as a triage based on a combination of HPV16/18 and cytology.

## Discussion

There is increasing evidence that cervical precancer arises primarily from persistent hrHPV infection in concert with reasonably well-understood abnormal epigenetic events. Host cells can respond to HPV infection with a cascade of defense mechanisms such as induction of interferon pathways, increased APOBEC-related DNA editing, and increased methylation. Such activity can get out of control and result in genome integrity problems, which in pluripotent cells have dangerous effects. Since the defense mechanisms are unable to subdue persistent HPV infections in a timely manner, they may instead participate in concert with continued over-expression of the HPV E6 and E7 oncoproteins as drivers towards cancer [28, 29, 41].

DNA methylation is a particularly tractable feature of the epigenetic machinery because patterns of CpG methylation are measurable as signatures and are stably retained during mitotic cell division. This feature encouraged us to explore the clinical utility of the S5 DNA methylation classifier as a triage test in our study of women with abnormal cytology and/or HPV16 and HPV18 infections. An important finding of our study is that the S5 classifier, at a modified cutoff of 3.7 (more favorable for women already triaged by cytology or HPV16/18 testing), was able to detect 62.0% of CIN2+, 70.3% of CIN3+, and all cancers in our study. In particular, S5 was able to provide important additional information for women with discrepant results between HPV16/18 and cytology. We believe that a combined triage approach that is cost-effective, with good specificity and a sensitivity of greater than 60% for CIN2/3, is generally safe for use in Mexico and also in women residing in low- and middle-income countries (LMIC), because of two important considerations: firstly, it is not crucial to detect all precancers in the first screen because most of these lesions regress or progress quite slowly; however, cancers are not reversible, and in our study, all cancers were detected by S5. Furthermore, in two other studies, S5 was also shown to have a ~ 100% sensitivity for cervical cancer [28, 32]. Secondly, additional intensive follow-up of all triage test-negative women is a standard recommendation, meaning that most if not all cancers in development can be caught at an early stage when cure rates are high (> 95%) as long as the women alerted to follow-up adhere to recommendations. A pressing priority in Mexico and indeed in most LMIC is to not overwhelm colposcopy clinics. A workload increase in these clinics could lead to increasingly poor outcomes and potentially to increases in cancer due to hurried and inadequate assessment of women, who may be given false-negative diagnoses or who may be lost to follow-up [42, 43]. Our results support a proposal to create enhanced risk scores that combine methylation of target genes with current cytology and HPV16/18 triage testing options for hrHPV screen-positive women.

Another major finding of our study is confirmation of the diagnostic utility of S5 seen in two earlier validation studies, a large screening study in the UK and a randomized control trial in Canada [28, 32]. In the hrHPV-based screening population from the UK, using a cutoff of 0.8 for S5, the AUC obtained for CIN2+ was 0.78 (95% CI 0.69–0.88), similar to the AUC of 0.75 (95% CI 0.69–0.8) seen in our FRIDA population. The 0.8 cutoff used in the UK population gave a higher sensitivity but a lower triage specificity compared with our S5 cutoff of 3.7. In previous studies of S5 performance, the sensitivity for cancer at the suggested triage cutoff of 0.8 was 100%. In our study, we showed that even at a cutoff of 3.7, all cancers were detected. Similar data have also been shown in other studies [28, 32], which indicates that few if any cancers would be missed when employing DNA methylation signatures for triage, even with a rather high S5 cutoff of 3.7 or perhaps higher.

Most lesions resulting from an infection with hrHPV eventually regress and only a very small number (< 5%) ever go on to invasive cancer in the absence of screening [3, 6]. To decrease costs and to avoid a large amount of unnecessary treatment, it is of great importance to identify hrHPV-positive women who are most likely to develop truly precancerous high grade CIN and to clearly separate them from women with CIN2 and CIN3 who will not develop cervical cancer at all or at least not over several decades [9, 14, 44]. Previous studies have proposed classifiers based on the methylation of HPV types (classifiers S1, S2 [22], and S4 [30]) and or human genes [40].

The S5 classifier was positive in 27% of our selected controls, but it is worthwhile to recall that these were women with either HPV16/18 infection or an abnormal cytology who were not subsequently diagnosed with CIN2+. Previous studies have observed that DNA methylation can predict the persistence of HPV infection of different viral types [22–24, 45]. Thus, it can be suggested that women, for whom a first colposcopy evaluation detected no significant lesions, could be carefully evaluated in follow-up visits to document quantitative changes in methylation consistent with the clearance or progression of disease.

Our results are applicable to methylation triage of women who are discrepantly positive for HPV16/18 and/or cytology ASCUS+; however, we were not able to assess if S5 could be used for triage of all hrHPV-positive women. The use of S5 could reduce colposcopy rates by up to 50% without a loss of sensitivity to detect cancer, which would be a big saving for any LMIC, not just because of lower financial costs but because of overall lower clinical burden. Furthermore, as shown by our results on women with concordant positive results for both HPV16/18 and cytology, the S5 test may also be useful for women who had a normal colposcopy. In this situation, a negative methylation result (S5 = 0) would provide reassurance against missed disease, especially if there is a high risk of loss to follow-up. The new cutoff proposed and different values of high methylation need to be validated in the setting of LMIC to estimate the clinical utility in populations with a similar context of income and disease prevention strategies to estimate the risk of cervical cancer.

The use of our particular triage population underestimates the number of potential cases of CIN2+ attributable to non-HPV16/18 hrHPV types; however, many of these women may be identified on follow-up. We consider that an introduction of S5 DNA methylation testing for routine use in self-validated expert clinical diagnostics laboratories is a reasonable course and, in some cases, could be undertaken quite quickly, being advantageous to further triage of women with HPV16/18 and/or abnormal cytology results. Successful implementation would allow colposcopy clinics to see only the highest-risk women and thereby attain higher quality and better manage overtreatment. Excessive burdening of clinical services and overtreatment of women are negative features of medical practice today due to an increasing flood of new women identified in expanded national HPV screening programs [46]. Based on practical experience from our laboratory, the cost of an S5 DNA methylation test is not more than a routine HPV screening and reflex genotyping. Thus, the S5 test could be applied to a minority of the women, and incremental costs could be balanced against the expected 30 to 50% reduction in colposcopy referral costs. Also, of note, the three cancers were detected by both HPV16/18 testing and the S5 test, but all of them were missed by cytology (Table 3). If confirmed by larger studies, this observation may allow for a generalized rapid reduction in cervical cancer in LMIC, which have been particularly poorly served by cytology screening over the past 50 years. Expected large-scale cervical cancer incidence reductions from implementation of the prophylactic HPV vaccine are still many decades away, and it is prudent to take more preventative actions in the meantime.

Our study has some important strengths including that it is the largest screening multi-triage study of cervical cancer in an LMIC. All primary clinical and laboratory procedures (screening, triage, colposcopy, and histopathological assessments) were done completely within Mexico. We selected all available cases and three matching controls, the latter specimens being selected at the same time and diagnosed in the same corresponding months as the cases. The accuracy of the colposcopic evaluation in FRIDA was very high and was not typical of Mexico, LMICs, or even developed countries such as the USA and Europe because it was performed by specially trained experts and also did not depend only on the visual expertise of the colposcopists. At least one biopsy was taken from each quadrant of the cervix in all women to reduce verification bias. Additionally, the histopathological evaluation and diagnostic confirmation of the biopsies and/or endocervical samples of all women included in our study were reviewed by a panel of pathologists, including an international expert. Moreover, all methylation measurements were done by blinded laboratory staff, including a scientist from Mexico (RH), with the aim of avoiding differential information bias.

## Conclusions

The S5 methylation classifier provides good sensitivity, specificity, and positive predictive value as a triage test for colposcopy referral in Mexican women with HPV16/18+ and/or ASCUS+, especially if CIN2/3 with low methylation do not progress, which is a subject of ongoing studies. We believe that our study and the existing literature on this topic support the use of methylation biomarkers in the triage of hrHPV-positive women within cervical cancer prevention programs in both highly developed settings and in LMICs. S5 methylation triage can reduce the numbers of needed colposcopies by an additional 30 to 50% as compared to triage by cytology and HPV16/18 genotyping while detecting all cancers. Such steps would greatly reduce existing costs and allow healthcare staff to better focus on the women at real risk of cervical cancer.

## Methods

### Study population

We performed a nested case-control analysis in a subset of hrHPV-positive women participating in the FRIDA screening study, all of whom underwent colposcopy (Fig. 1). Details of the study methods have been described previously [47]. Briefly, FRIDA is a population-based study that recruited 36,212 women from a target population of close to 60,000 women aged 30–64 years, between April 2013 and February 2016. Women were users of public health services, who attended the Cervical Cancer Screening Program in 100 primary health care facilities in the state of Tlaxcala, Mexico. The main aim of FRIDA was to evaluate the performance of different triage tests for hrHPV-positive women. Women who were pregnant at the time of recruitment or had a prior hysterectomy were excluded. Informed consent was required for women to participate in the study. The population sampling for this nested case-control study was selected from a pool of women attending colposcopy because they were either singly positive or positive for both cytology and HPV16/18 between April 2013 and July 2015, when 29,759 women had been enrolled. Women who were infected by HPV types other than HPV16 or HPV18 who did not have cytological abnormalities were excluded for this analysis.

FRIDA is registered in ClinicalTrials.gov, number NCT02510027, and was approved by the Institutional Review Boards (IRBs) of the participating institutions: National Public Health Institute (INSP) [1094], Tlaxcala State Ministry of Health [SS.DECI-OI-13/12], and the Mexican regulatory agency COFEPRIS [CAS/OR/01/CAS/123300410C0044-3578/2012].

### Selection of cases and controls

The nested case-control triage study considered hrHPV-positive women who attended for colposcopy as a result of a positive test by HPV16/18 (*n* = 586) or a cytological diagnosis of atypical squamous cells of undetermined significance or worse (ASCUS+) (*n* = 277). HPV testing for 13 high-risk HPV types and HPV66, followed by HPV16/18 genotyping, were performed by the Cobas® 4800 DNA test (Roche, Pleasanton, CA, USA) as described below. Endocervical curettage was performed for all women referred for colposcopic evaluation, during which one biopsy was also collected from what the colposcopist suspected to be the most abnormal zone of each quadrant. Evaluation of all histological samples was conducted by a standardized panel of pathologists. If two pathologists agreed in their diagnosis, this was the final conclusion. If the diagnoses were discordant, an additional interpretation was made by a third expert pathologist. In this case, the assigned diagnosis was based on the agreement of two out of three pathologists or, if totally discordant, on the result given by the expert pathologist.

Cases and controls were selected based on the histological status. All women diagnosed with CIN2, CIN3, or invasive cervical cancer were defined as cases. For each case identified, we selected three age-matched controls: one control diagnosed with low-grade cervical intraepithelial neoplasia (CIN1) and two controls with no evidence of any cervical intraepithelial lesion (NEG). Controls were selected randomly within a 2-month time frame of the case diagnosis.

### Collection and shipment of cervical samples

DNA methylation assays were performed on an aliquot of cervical samples obtained from the screening visit. In this visit, a first cervical sample was collected in a SurePath (Becton Dickinson, Sparks, MD, USA) vial and used for the cytology procedure. Cervical samples for HPV and methylation testing were collected as a second sample and placed in a vial containing ThinPrep® preservative (Hologic, Inc., Bedford, MA). Both samples were collected using a Cervex-Brush® (Rovers®) in the screening visit. The samples were temporarily stored at room temperature at the health center until they were delivered to HPV laboratory facilities where aliquots were made and kept frozen at − 70 °C. The samples were shipped on dry ice to the Molecular Epidemiology Laboratory of the Wolfson Institute of Preventive Medicine (Queen Mary University of London, UK) for analysis.

### HPV detection

The samples were tested for hrHPV using the Cobas® 4800 HPV test (Roche Molecular Systems, Pleasanton, CA). This system extracted DNA and amplified by PCR using specific primers to human beta globin and hrHPV. HPV16 and HPV18 are identified individually and other HPV types (16, 18, 31, 33, 35, 39, 45, 51, 52, 56, 58, 59, 66, and 68) were detected as a pool.

Since the Cobas® assay does not give individual typing of HPV31 and HPV33, the prevalence of these two HPV types was estimated by the detection of amplicons specific for these two HPV types in the PCR reactions (HPV31L1 and HPV33L2) leading up to the methylation assays.

### Methylation assays

For methylation assays, the DNA was extracted from cervical samples using a QIAamp mini kit (Qiagen, Hilden, Germany) following the manufacturer’s instructions. Subsequently, the DNA was quantified by UV absorption using a NanoDrop spectrophotometer. An aliquot of 500 ng of DNA underwent bisulfite conversion in which unmethylated cytosines were converted to uracil using the EZ DNA methylation kit (Zymo Research, Irvine, CA) following the manufacturer’s instructions. This kit is designed to reduce degradation and minimize the loss of DNA. The DNA was eluted in 20 μl of Elution Buffer.

After DNA conversion, the PyroMark PCR Kit (Qiagen) was used to amplify the following CpG sites: HPV16 L1 (6367 and 6389), HPV16 L2 (4238, 4247, 4259, 4268, and 4275), HPV18 L2 (4256, 4261, 4265, 4269, 4275, and 4282), HPV31 L1 (6352, 6364), HPV33 L2 (5557, 5560, 5566, and 5572), and human gene *EPB41L3* (425, 427, and 438). PCR products were pyrosequenced using a PyroMark Q96 ID instrument (Qiagen), and the proportions of cytosine and thymine were quantified for each CpG site. The laboratory was blinded to the cytology and histology results. The procedures and quality control of these experiments have been reported previously [23, 27].

### Data analysis

Socio-demographic, lifestyle, and sexual behavior of cases and controls were summarized as means or proportions. We computed the specific prevalence of HPV types (16, 18, 31, and 33) within our study population. Differences across all variables between cases and controls were tested using a two-sided, difference of proportions by a binomial test or difference of medians by a Mann-Whitney *U* test. Our primary goal was to assess the clinical performance of the S5 classifier. This score was calculated using the average methylation values of all the target regions using the following standardized equation [31]:

*S5* = EPB41L3*(30.9) + HPV16L1.3*(13.7) + HPV16L2*(4.3) + HPV18L2*(8.4) + HPV31L1*(22.4) + HPV33L2*(20.3)

We created boxplots to illustrate the distribution of the S5 classifier according to the histopathological diagnosis of the lesions (NEG, CIN1, CIN2, CIN3, and invasive cervical cancer (CC)). We used the Mann-Whitney *U* test for comparing S5 differences between different disease categories and the Cuzick test for trend to determine if methylation increased significantly as a function of greater histology result.

In order to maximize the sensitivity and specificity of the S5 score after the triage test (HPV16/18 and cytology) we calculated receiver operating characteristic curves (ROC) to estimate areas under the curve (AUC) and to obtain a new cutoff value that best discriminated hrHPV-positive women with CIN2+ (cases) from controls who did not have histologic abnormalities, in order to maximize the sum of sensitivity and specificity of the S5 score among our already-triaged samples. Based on this new cutoff value, we set a disease endpoint of CIN2+ to estimate sensitivity, specificity, positive predictive value (PPV), and negative predictive value (NPV); the PPVs were also adjusted for CIN2+ and CIN3+ prevalence (10.0% and 6.8% respectively) using the following formula: PPV = (Sn × Pr)/((Sn × Pr) + (1 − Sp) × (1 − Pr)), where Sn is sensitivity, Sp is specificity, and Pr is the CIN2+ or CIN3+ prevalence. A ROC curve was also computed for CIN3+ versus NEG/CIN1. We compared the performance of S5 with HPV16/18 detection and cytology to identify CIN2+ using crude estimators because it was not possible to adjust for the bias created by not referring all hrHPV positive women to colposcopy; in effect, HPV16/18 diagnostic performance was assessed versus a comparator composed of HPV16/18 plus cytology ASCUS+; similarly, ASCUS+ cytology performance was compared to the same composite comparator. A second analysis was done using the established 0.8 cutoff validated earlier in a UK screening population for detection of CIN2+ and CIN3+. All *p* values were estimated as two sided, with a confidence interval of 95%. Statistical analyses were performed using STATA software v.14.0 (StataCorp LP, College Station, TX).

## Data Availability

The full datasets generated from and/or analyzed during the current study are not publicly available due to the need to protect participant confidentiality; however, selected anonymized data can be made available by the FRIDA steering committee. Inquiries should be communicated to the corresponding author who will work with other authorized members of the FRIDA team to consider all sufficiently specified and reasonable requests.

## Abbreviations

ASCUS: Atypical squamous cells of undetermined significance
AUC: Area under the curve
CC: Cervical cancer
CI: Confidence interval
CIN1: Cervical intraepithelial neoplasia grade 1
CIN2: Cervical intraepithelial neoplasia grade 2
CIN2+: Cervical intraepithelial neoplasia grade 2 or higher
CIN3: Cervical intraepithelial neoplasia grade 3
CpG: Cytosine-phosphate-Guanine site
hrHPV: High-risk human papillomavirus
HSIL: High-grade squamous intraepithelial lesions
L1: Late region 1
L2: Late region 2
LSIL: Low-grade squamous intraepithelial lesions
NEG: Negative for cervical intraepithelial neoplasia
NPV: Negative predictive value
PPV: Positive predictive value
ROC: Receiver operating characteristic
